# Development of Pathological Diagnostics of Human Kidney Cancer by Multiple Staining Using New Fluorescent Fluolid Dyes

**DOI:** 10.1155/2014/437871

**Published:** 2014-06-03

**Authors:** Dilibaier Wuxiuer, Yun Zhu, Takunori Ogaeri, Keiji Mizuki, Yuki Kashiwa, Kentaro Nishi, Shin-ichiro Isobe, Tei-ichiro Aoyagi, Ryoiti Kiyama

**Affiliations:** ^1^Biomedical Research Institute, National Institute of Advanced Industrial Science and Technology (AIST), 1-1-1 Higashi, Tsukuba, Ibaraki 305-8566, Japan; ^2^Department of Nanoscience, Faculty of Engineering, Sojo University, 4-22-1 Ikeda, Kumamoto 860-0082, Japan; ^3^Department of Applied Chemistry and Biochemistry, Faculty of Engineering, Kyushu Sangyo University, 2-3-1 Matsukadai, Higashi-ku, Fukuoka 813-8503, Japan; ^4^Ibaraki Medical Center, Tokyo Medical University, 3-20-1 Ami, Inashiki, Ibaraki 300-0395, Japan

## Abstract

New fluorescent Fluolid dyes have advantages over others such as stability against heat, dryness, and excess light. Here, we performed simultaneous immunostaining of renal tumors, clear cell renal cell carcinoma (RCC), papillary RCC, chromophobe RCC, acquired cystic disease-associated RCC (ACD-RCC), and renal angiomyolipoma (AML), with primary antibodies against Kank1, cytokeratin 7 (CK7), and CD10, which were detected with secondary antibodies labeled with Fluolid-Orange, Fluolid-Green, and Alexa Fluor 647, respectively. Kank1 was stained in normal renal tubules, papillary RCC, and ACD-RCC, and weakly or negatively in all other tumors. CK7 was positive in normal renal tubules, papillary RCC, and ACD-RCC. In contrast, CD10 was expressed in renal tubules and clear cell RCC, papillary RCC, AML, and AC-RCC, and weakly in chromophobe RCC. These results may contribute to differentiating renal tumors and subtypes of RCCs. We also examined the stability of fluorescence and found that fluorescent images of Fluolid dyes were identical between a tissue section and the same section after it was stored for almost three years at room temperature. This indicates that tissue sections can be stored at room temperature for a relatively long time after they are stained with multiple fluorescent markers, which could open a door for pathological diagnostics.

## 1. Introduction

Owing to the increased availability of diagnostic markers for pathological evaluation of cancer, there has been an increased demand for staining valuable specimens with multiple and combinational markers. There have been approaches based on double, triple, and even quadruple staining of specimens with the respective numbers of markers [1–5]. However, there has been difficulty in putting such staining methods into practice due to various problems, such as the quality of methods, and the stability and biological relevance of markers [6]. When colorimetric staining is used, such as that with alkaline phosphatase- or horseradish peroxidase-conjugated antibodies, multiple markers are hard to differentiate visually. In contrast, when multiple fluorescent markers are used for staining, stained specimens cannot be stored for a long time due to the poor stability of fluorescent dyes. Thus, a system for multiple staining using stable fluorescent dyes is crucial to develop a new diagnostic protocol for the pathological examination of cancer. A pathological application was explored previously with a new fluorescent dye, Fluolid-Orange [7]. Another Fluolid dye, Fluolid-Green, is now available and these Fluolid dyes show strong fluorescence even in the solid state, large Stokes shifts, and stability against dryness, heat, and excess light [8] and are thus ideal for long-term storage of stained specimens.

Kidney and urinary tract cancers accounted for 8,334 deaths in 2012 in Japan, roughly 2% of all cancers [9]. Renal cell carcinoma (RCC) is the most common type of kidney cancer and is classified into five histologic subtypes, clear cell (70–80%), papillary (10–15%), chromophobe (3–5%), collecting duct (1%), and unclassified (1%) RCC [10]. A quarter of patients with RCC will develop locally advanced or metastatic diseases and a third of patients with localized disease at presentation will have recurrence thereafter [11, 12]. Since the malignant nature and therapeutic response to recent molecular targeting agents differ among the histological subtypes of RCC, it is critical to make a correct diagnosis of renal tumors. For example, the 5-year survival of RCC is estimated to be approximately 62% for all stages, while that of distant metastasis decreases to 10% [13]. Furthermore, a number of pathological markers have been developed to improve the poor survival of metastatic RCC [14]. Therefore, detection of cytopathological markers simultaneously using multiple fluorescent dyes would be valuable in the pathological diagnosis to differentiate renal tumors and cancer subtypes. When a clinician has to make a decision using pathological specimens obtained by needle biopsy, for example, detection of several cytopathological markers simultaneously would be very useful. Furthermore, it would be an advantage to be able to reexamine tissue sections again after long-term storage. Thus, the stability of fluorescent dyes is quite important.

In order to develop a new technique for immunohistochemical staining in the pathological diagnosis of cancer, we examined here tissue sections containing human renal tumors by means of quadruple staining using antibodies labeled with two Fluolid dyes, Fluolid-Green and Fluolid-Orange, in combination with Alexa Fluor 647 and 4′,6-diamidino-2-phenylindole (DAPI). Antibodies against Kank1, cytokeratin 7 (CK7), and CD10 proteins were used as the primary antibodies and Fluolid-conjugated IgG (Kank1 and CK7) and Alexa Fluor 647-conjugated IgG (CD10) were used as the secondary antibodies to detect the primary antibodies. The gene for Kank1 (*Kank1*) was found to be a tumor suppressor gene and its expression was decreased or lost in renal tumors [15]. CK7 and CD10 have been used in the histologic diagnosis of renal tumors [16–18]. CD10, or neprilysin, is a cell-surface glycoprotein expressed in specific subtypes of renal tumors and has zinc-dependent metalloprotease activity that degrades small secreted peptides, such as the amyloid beta peptide [18]. CK7 is a type II keratin expressed in simple glandular epithelia and in transitional epithelium. CK7 has been used to differentiate chromophobe and papillary RCCs [17].

## 2. Materials and Methods

### 2.1. Reagents

A rabbit anti-human cytokeratin 7 (CK7) antibody was purchased from Funakoshi (Tokyo, Japan), a goat anti-human neprilysin (CD10) antibody from R&D Systems (Minneapolis, MN), and donkey anti-mouse IgG and donkey anti-rabbit IgG from Jackson ImmunoResearch (West Grove, PA). Alexa Fluor 647-conjugated donkey anti-goat IgG was purchased from Life Technologies (Carlsbad, CA). Fluolid-Orange and Fluolid-Green were purchased from Cosmo Bio (Tokyo, Japan).

### 2.2. Immunohistochemistry

IgG was labeled with each Fluolid dye using a Fluolid-W protein/antibody labeling kit (International Science Technology, Fukuoka, Japan) according to the manufacturer's instructions. Briefly, IgG was dissolved into 0.2 M sodium bicarbonate buffer (pH 8.3), mixed with a Fluolid dye (dissolved in DMSO), and incubated for 2 hr at room temperature. Unreacted dye was removed with a NAP-5 column (GE Healthcare Japan, Tokyo, Japan).

Paraffin-embedded specimens from RCC patients were obtained upon written informed consent after approval from the ethics committees of Tokyo Medical University and the National Institute of Advanced Industrial Science and Technology. Specimens were deparaffinized by washing three times with fresh xylene for 5 min and then washing with graded ethanol, followed by washing twice with phosphate-buffered saline (PBS). For retrieving antigens, samples were boiled in 10 mM citrate buffer (pH 6.0) for 10 min using a microwave oven as reported previously [19]. Blocking was performed with 5% (v/v) donkey serum in 0.3% Triton-X 100/PBS, followed by washing twice with PBS. An anti-Kank1 monoclonal antibody [20] diluted 1 : 50 with PBS, an anti-CK7 polyclonal antibody diluted 1 : 100 with PBS, and an anti-CD10 polyclonal antibody diluted 1 : 100 with PBS were used as the primary antibodies. The samples were incubated with the antibodies overnight at 4°C and then washed three times with PBS. Fluolid-Green-conjugated anti-mouse IgG (1 : 100) and Fluolid-Orange-conjugated anti-rabbit IgG (1 : 100) and Alexa Fluor 647-conjugated anti-goat IgG (1 : 500) were used as the secondary antibodies. The samples were washed with PBS and stained for 10 min with 0.2 *μ*g/mL DAPI (Invitrogen, Carlsbad, CA). The samples were observed using a confocal laser scanning microscope (LSM 510 META; Carl Zeiss, Oberkochen, Germany) under the following conditions: 488 nm excitation/500–560 nm emission for Fluolid-Green; 488 nm/580–650 nm for Fluolid-Orange; and 633 nm/LP650 nm for Alexa Fluor 647. Images were acquired using LSM510 ver. 3.2 software (Carl Zeiss) and processed with Photoshop 7.0 (Adobe Systems, San Jose, CA).

## 3. Results

### 3.1. Immunostaining of Renal Tumors with Fluolid-Labeled Antibodies

To examine the usefulness of Fluolid dyes in multiple immunostaining, we performed immunostaining using antibodies labeled with two different Fluolid dyes (Fluolid-Green and Fluolid-Orange). Alexa Fluor 647 was used as the third fluorescent dye and also as a reference. Antibodies against Kank1, CK7, or CD10 proteins raised in mice, rabbits, or goats, respectively, were used as the primary antibodies and fluorescently labeled IgGs for respective species were used as the secondary antibodies. DAPI was included as the fourth dye to localize nuclei. We first performed single-dye immunostaining of kidney tissues to detect Kank1, CK7, or CD10, respectively, and confirmed that the respective fluorescence images did not overlap (data not shown). We then immunostained normal mouse kidney tissues with multiple markers (data not shown) and then applied the system to immunostain human renal tumors.

**Figure 1:**
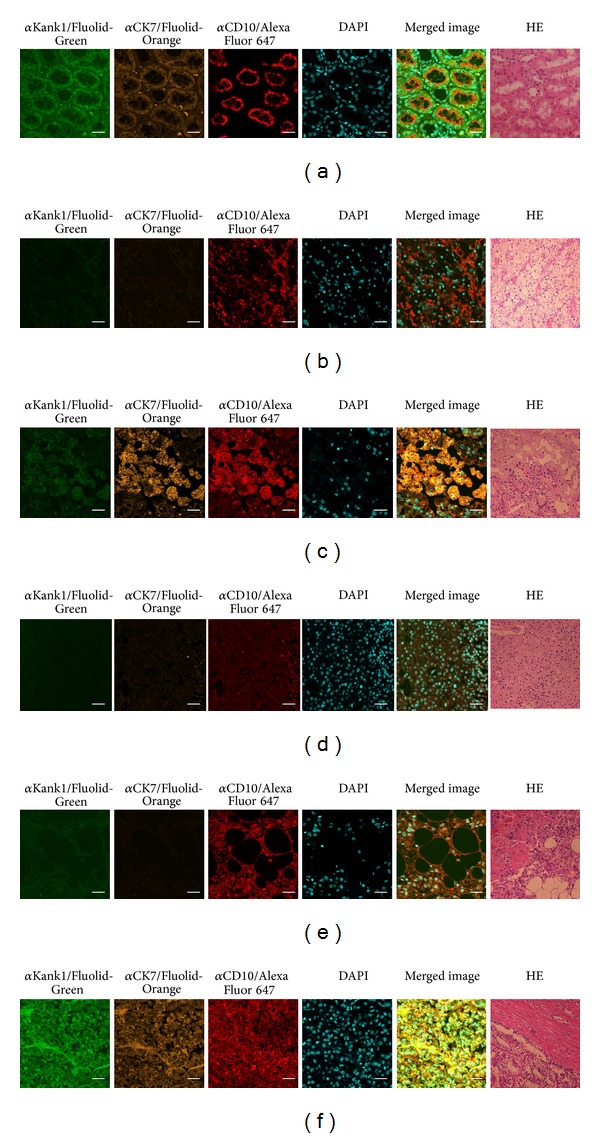
Immunostaining of renal tumors. Antibodies against Kank1 (*α*Kank1), CK7 (*α*CK7), and CD10 (*α*CD10) were used as the primary antibodies, and Fluolid-Green-conjugated donkey anti-mouse IgG, Fluolid-Orange-conjugated donkey anti-rabbit IgG, and Alexa Fluor 647-conjugated donkey anti-goat IgG were used, respectively, as the secondary antibodies. DAPI was used to stain nuclei and tissues were histopathologically examined by hematoxylin and eosin (HE) staining. Results of immunostaining of normal tissue (a), clear cell RCC (b), papillary RCC (c), chromophobe RCC (d), renal angiomyolipoma (e), and acquired cystic disease-associated RCC (f) are shown. Note that the parts shown by HE staining in cancer do not match with those shown by immunostaining. Image magnification: ×40 for immunostaining in (a)–(f) and HE staining in (a); ×20 for HE staining in (b)–(f). Bar = 20 *μ*m.

The results of simultaneous staining of renal tumors with multiple markers labeled with Fluolid dyes are shown in Figure 1. In normal kidney tissue, Kank1 and CK7 were expressed in the cytoplasm of renal tubular cells, while CD10 was expressed in the membrane of renal tubular cells (Figure 1(a)). We applied this quadruple staining system to examine cancerous areas of clear cell RCC (Figure 1(b)), papillary RCC (Figure 1(c)), chromophobe RCC (Figure 1(d)), AML (Figure 1(e)), and acquired cystic disease-associated RCC (ACD-RCC or RCC in dialysis patients) (Figure 1(f)). In tumor sections, Kank1 was stained in papillary RCC and ACD-RCC and weakly or negatively in all other tumors. CK7 was also positive in papillary RCC and ACD-RCC. CD10 was expressed in clear cell, papillary and ACD-RCCs, and AML and weakly in chromophobe RCC. These results are summarized in Table 1.

### 3.2. Stability of Fluorescence in Pathological Specimens

**Figure 2:**
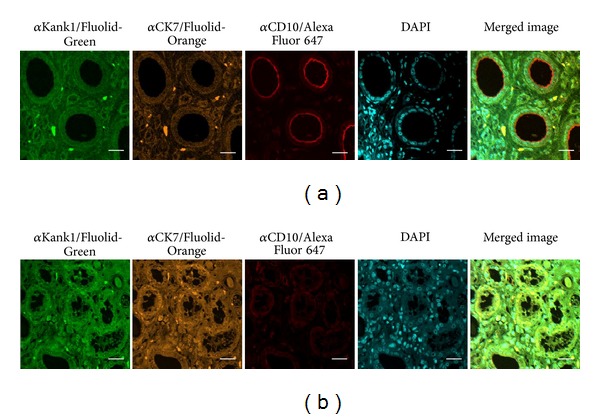
Stability of fluorescent dyes in histopathological sections. Fluorescent images were examined for a tissue section before (a) or after (b) the storage at room temperature for almost three years. A normal human kidney tissue section was subjected to quadruple staining with three different Fluolid dyes and DAPI. Antibodies against Kank1, CK7, and CD10 were used as the primary antibodies, and Fluolid-Green-conjugated anti-mouse IgG, Fluolid-Orange-conjugated anti-rabbit IgG, and Alexa Fluor 647-conjugated anti-goat IgG were used as the secondary antibodies. Bar = 20 *μ*m.

We also examined the stability of Fluolid dyes in a section that was stored at room temperature for almost three years (Figure 2). A normal human kidney tissue section observed previously (Figure 2(a)) was observed again two years and 11 months later (Figure 2(b)). The result showed that fluorescent images of Fluolid dyes were identical between the original and the section years later, while Alexa Fluor 647 became very weak.

## 4. Discussion

In this study, we examined whether Fluolid dyes are useful for pathological diagnosis and showed examples of quadruple staining of human renal tumors using Fluolid dyes (Fluolid-Green and Fluolid-Orange) along with Alexa Fluor 647 and DAPI. Antibodies against Kank1, CK7, and CD10 were used as the primary antibodies and fluorescently labeled IgGs were used as the secondary antibodies. To effectively separate the images from different fluorescent dyes, it was necessary to set up an appropriate set of fluorescence filters. We first examined the assay system including the filter set by evaluating whether the images were completely separated, observing the images after single, double, or triple staining using all combinations of Fluolid dyes, Alexa Fluor 647, and DAPI (see Section 2). Then, we made an assay system for quadruple staining using all fluorescent dyes.

Using this system, we performed quadruple staining of clear cell RCC, papillary RCC, chromophobe RCC, AML, and ACD-RCC (Figure 1). Seeing the differences among these subtypes of renal tumors is one of the most challenging differential diagnoses [16]. The overlapping nuclear, cytoplasmic, and architectural characteristics among these five tumor subtypes have been well documented by hematoxylin and eosin (HE) staining [16, 17]. In addition, we found that multiple immunohistochemical markers with differential expression in these renal tumors are available (Figures 1(b)–1(f)).

As summarized in Table 1, we were able to differentiate the above subtypes of renal tumors by analysis of the differential expression of these markers. Here, Kank1 was pathologically examined for the first time in RCC subtypes, where Kank1 was negative in clear cell and chromophobe RCCs, and in AML, but positive in papillary RCC and ACD-RCC (Table 1). Kank1 was found as a tumor suppressor of clear cell RCC [15], and the locus (9p24.3) is frequently deleted in clear cell RCC [21]. Meanwhile, CK7 was positive in papillary RCC but negative in chromophobe RCC. CK7 is localized in the cytoplasm of most papillary RCC, collecting duct RCC and urothelial carcinoma, but it is also positive in other tumors [18]. While CK7 has been reported to be positive in many cases of chromophobe RCC, 91% for example, [17], there were reports of relatively low frequencies, such as 66% [22], suggesting the contribution of genetic variations among patients with chromophobe RCC. Notably, there is a possibility that its diffuse and membranous staining may need further improvement in fluorescent detection, especially in enhancing the brightness of images. AML is thought to be a benign tumor, and, thus, surgical treatment is not necessary if the tumor is correctively diagnosed by computed tomography (CT) or ultrasonography. However, it is sometimes difficult to differentiate it from malignant tumors when the tumor is small or there is no fatty component [23]. Therefore, it may be helpful to distinguish AML from other malignant tumors in a small specimen. In our study, CK7 was negative in AML, as reported previously [24]. It was reported that CK7 is negative in ACD-RCC [25], although it was positive in our study (Table 1).

CD10 is a surface glycoprotein identified in a variety of healthy cells, where it hydrolyzes peptide bonds and decreases the cellular response to local peptide hormones. In the normal kidney, CD10 is strongly expressed at proximal tubular cell brush borders [18]. CD10 is strongly and diffusely expressed in renal cell neoplasms derived from proximal tubules in clear cell RCC and may be present in papillary RCC, usually not in a diffuse manner but in a luminal pattern. CD10 is also expressed in vascular cells of AML [26] and in ACD-RCC [27], but is usually negative in chromophobe RCC [28]. Note that CK7 and CD10 are sometimes used in combination to differentiate subtypes of RCC [16].

We also examined the stability of fluorescently labeled markers on a section that was stored at room temperature for almost three years (Figure 2). The fluorescent images of Fluolid dyes were identical between the original and the section years later, while that of Alexa Fluor 647 became weak. Therefore, Fluolid dyes are advantageous for long-term preservation of stained histopathological sections.

Multiple immunoenzyme staining systems have been developed by modifying standard chromogen-based immunoenzyme techniques. Double staining systems were developed to achieve maximum color contrast between red (such as 3-amino-9-ethyl-carbazole) and brown (such as 3,3′-diaminobenzidine or DAB) by unmixing spectral images [29]. However, colorimetric separation of signals among colocalizing markers, and between markers and endogenous pigments, is almost impossible, and so, only double staining, or triple staining for limited cases [29], can be applied. In contrast, a variety of images of different markers can be separated with good contrast (high signal/noise ratios) and sensitivity (low background signals) in immunofluorescent techniques. However, the storage of stained specimens is mostly limited to days/weeks due to poor stability of fluorescent dyes. Here, we provided a double staining system with stable fluorescent dyes. However, this is only a progress in the process to develop a future immunostaining system where the third stable fluorescent dye with a complete set of markers and automated, less expensive devices are available. A new Fluolid dye compatible with Alexa Fluor 647 is now under development to provide an immunohistochemical system with three stable markers.

In this study, we provided examples of quadruple staining of sections of human renal tumors using Fluolid dyes, Alexa Fluor 647, and DAPI. In conclusion, immunostaining with multiple fluorescently labeled markers will improve the classification of renal tumor subtypes using limited amounts of samples, which may be of particular help for pathologists and clinicians. Furthermore, Fluolid dyes will enable long-term preservation of stained histopathological sections. Hopefully, this multiple staining method will improve the accuracy of the diagnosis of various diseases.

## Figures and Tables

**Table 1:** Summary of immunostaining of Kank1, CK7, and CD10 in renal tumors.

Marker	Renal tumor				
	Clear cell RCC	Papillary RCC	Chromophobe RCC	AML	ACD-RCC
Kank1	−	+	−	−	+
CK7	−	++	−	−	+
CD10	++	+	±	+	+
